# Printing 3D Hydrogel Structures Employing Low-Cost Stereolithography Technology

**DOI:** 10.3390/jfb11010012

**Published:** 2020-02-22

**Authors:** Leila Samara S. M. Magalhães, Francisco Eroni Paz Santos, Conceição de Maria Vaz Elias, Samson Afewerki, Gustavo F. Sousa, Andre S. A. Furtado, Fernanda Roberta Marciano, Anderson Oliveira Lobo

**Affiliations:** 1 LIMAV–Interdisciplinary Laboratory for Advanced Materials, BioMatLab, Materials Science and Engineering Graduate Program, UFPI–Federal University of Piauí, Teresina 64049-550, Brazil; leilassmoreira@hotmail.com (L.S.S.M.M.); franciscoeroni@gmail.com (F.E.P.S.); salesandre7@gmail.com (A.S.A.F.); 2 Department of Physics, UFPI–Federal University of Piauí, Teresina 64049-550, Brazil; marciano@ufpi.edu.br; 3 Biomedical Engineering Graduate Program, University of Brazil, São Paulo 08230-030, Brazil; conceicaovazenf@hotmail.com; 4 Division of Engineering in Medicine, Department of Medicine, Harvard Medical School, Brigham & Women´s Hospital, Cambridge, MA 02139, USA; 5 Harvard-MIT Division of Health Science and Technology, Massachusetts Institute of Technology, MIT, Cambridge, MA 02139, USA

**Keywords:** stereolithography, bioprinting, 3D printing, hydrogels, GelMA, PEGDA

## Abstract

Stereolithography technology associated with the employment of photocrosslinkable, biocompatible, and bioactive hydrogels have been widely used. This method enables 3D microfabrication from images created by computer programs and allows researchers to design various complex models for tissue engineering applications. This study presents a simple and fast home-made stereolithography system developed to print layer-by-layer structures. Polyethylene glycol diacrylate (PEGDA) and gelatin methacryloyl (GelMA) hydrogels were employed as the photocrosslinkable polymers in various concentrations. Three-dimensional (3D) constructions were obtained by using the stereolithography technique assembled from a commercial projector, which emphasizes the low cost and efficiency of the technique. Lithium phenyl-2,4,6-trimethylbenzoyl phosphonate (LAP) was used as a photoinitiator, and a 404 nm laser source was used to promote the crosslinking. Three-dimensional and vascularized structures with more than 5 layers and resolutions between 42 and 83 µm were printed. The 3D printed complex structures highlight the potential of this low-cost stereolithography technique as a great tool in tissue engineering studies, as an alternative to bioprint miniaturized models, simulate vital and pathological functions, and even for analyzing the actions of drugs in the human body.

## 1. Introduction

Both industry and academia have expressed increased interest in the application of various printing technologies [1,2] because they offer great advantages such as faster production, easy access, better quality, cost-effective, tailormade design, minor waste generation, and allow large scale production [3]. Stereolithography has emerged as a more accurate method to design layer-by-layer light-sensitive hydrogels, rather than strips or droplets as employed in other bioprinting techniques [4,5]. The employment of this technology has promoted the emergence of micro-scale technologies and microfluidic systems, which are useful tools to overcome the challenges of creating artificial microvascular structures [6,7,8]. Consequently, these tools make bioprinting even more efficient in terms of versatility, detailing, and capacity to obtain structures with high spatial resolution [9]. Furthermore, hydrogels have been widely studied due to their unique properties such as biocompatibility and bioactivity. These materials may be natural or synthetic with an inherent crosslinking ability for biomedical applications, including tissue engineering scaffolds, artificial blood vessels, wound dressing, and drug delivery devices [10].

Stereolithography technologies have allowed effective advances in constructing detailed shapes with these hydrogel-based biomaterials made of complex and accurate media with different physical, chemical, and mechanical properties to study, create, and recover lost functional tissues and structures [11,12,13]. Nevertheless, challenges such as long manufacturing times and high cost still limit the application of this technology [14].

Furthermore, the selection of appropriate biomaterials for stereolithography intended for biomedical applications is crucial for engineering optimal and high quality printed structures [12]. Polyethylene glycol diacrylate (PEGDA) and gelatin methacryloyl (GelMA) have proven to be suitable candidates and have been mostly used to produce printed structures using stereolithography [15]. These light-sensitive and photocrosslinkable hydrogels have significant scientific interest due to their great advantages such as biocompatibility, hydrophilicity, and ability to promote various cellular functions, making them suitable for biomedical applications, tissue engineering, and regenerative medicine, pharmaceuticals, and cancer therapies [15,16]. In addition, these materials have been used as a cell culture platform to develop constructs or implants [17]. These types of hydrogels are not toxic and exhibit easy hydration, combine well with other biomaterials, and exhibit adhesive properties. Upon exposure to light, these materials achieve desirable functionality and are easily crosslinked suitable for various biomedical applications and three-dimensional (3D) constructions [16]. Moreover, a microfluidic digital mirror device (DMD) can be employed for micro-stereolithographic detail-rich constructs, which are required in tissue engineering applications [18,19]. This method enables 3D microfabrication of solid-state materials by creating images through computer-aided design programs (CAD) [20,21].

Generally, the stereolithography process consists of a concentrated UV light beam that shines on the liquid photopolymer, and in parallel, the CAD model is interpreted onto the surface of the liquid. This process initiates the synthesis of the first layer; subsequently, each additional layer is polymerized at the predetermined time to form layers until the intended object is fully fabricated. Stereolithography is widely used to manufacture physical models, such as human anatomy, for surgical procedures, and implants in medicine and dentistry [22,23].

Herein, we present custom-made low-cost stereolithography-based printing technology. The system consists of one commercial projector, laser radiation at 404 nm to promote the light polymerization of the liquid polymers towards a solid structure, and a computer, where the design is created. The models were produced from simple images and printed layer-by-layer. As a proof of concept, a wide range of various designed structures were printed.

## 2. Materials and Methods

### 2.1. Materials

The following chemicals were purchased from Sigma-Aldrich (St. Louis, MO, USA): PEGDA (Molecular Weight: 2000 Da), gelatin (Type A, 300 bloom from porcine skin), methacrylic anhydride (MA), polydimethylsiloxane (PDMS), polymethoxycarbonylmethylene (PMMA) polymers and phosphate buffered saline (PBS). Photoinitiator lithium phenyl-2,4,6-trimethyl benzoyl phosphinate (LAP, Allevi, Philadelphia, PA, USA) was used as a photoinitiator. GelMA was synthesized according to protocol [24]. Food dye (Mago, Sao Paulo, Brazil) was used to visualize the channels. All the chemicals were used without any further treatments or purifications.

### 2.2. Preparation of Formulations for Bioprinting Employing Stereolithography

*PEGDA hydrogel*: PEGDA (10 wt %) was dissolved in deionized water, followed by the addition of LAP (0.3 wt %). *GelMA hydrogel*: various concentrations of GelMA (7, 10, 12, 15, 20 and 25 wt %) were dissolved in PBS at 37 °C, followed by addition of the LAP (0.3 wt %).

### 2.3. Stereolithography Setup

**Figure 1:**
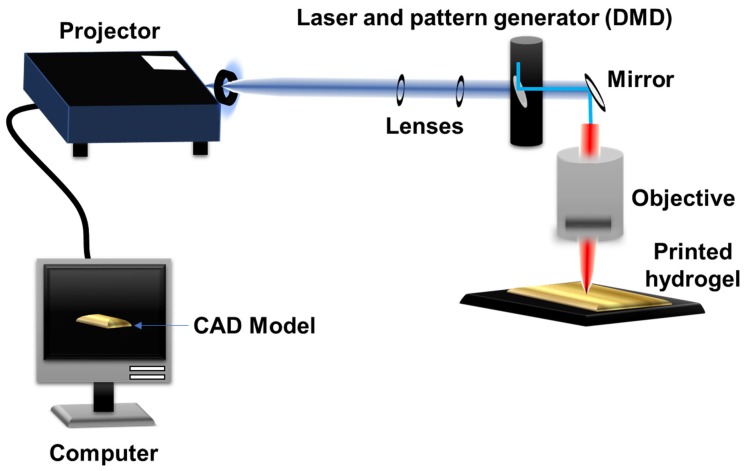
Schematic representation of the custom-made low-cost stereolithography with the various components including the computer, projector, lenses, digital micromirror device (DMD), mirrors, objective, and the printed hydrogel. The two lenses form a telescope, which controls the beam size without divergence. The computer program provides the computer-aided design (CAD) model to be printed with planned settings through the projector. The laser allows solidification of the hydrogels through crosslinking layer by layer.

Figure 1 depicts the custom-made stereolithography-based system for 3D structure fabrication. The computer program, Thinkercad software, was employed and the masks were built to form the 3D structures. To enable the slicing process, the 3D models were converted into 2D bitmap planes by using Cura software. The generated planes were designed by an Epson DLP type overhead projector. The light beam from the projector is a telescope formed by two lenses, which controls the beam diameter at the lens entrance and consequently the final size of the generated image. The process is conducted with a working distance of 2 cm. The photocrosslinking of PEGDA and GelMA was carried out using a coherent^®^ diode laser (404 nm, 500 mW cm^−2^). An energy meter (Thorlabs) was used to determine the light intensity and input beam. The PEGDA and GelMA solutions (as previously described) were placed under PDMS film and fixed in a PMMA mold. The 3D construction process of the printed layers were designed as biomimetic vascularization model structures, linear systems, and star shapes to evaluate the potential and resolution of the assembled technique. Different amounts of each hydrogel were placed on each layer constructed to cover the projection region of the images, and each layer was irradiated with laser light for up to 20 seconds, depending on the light opening and hydrogel composition for initiating the crosslinking processes. Constructions of 3 to 7 layers were obtained employing the devised technology.

### 2.4. Mechanical Testing

The mechanical properties of the hydrogels were characterized by an unconfined compression test. The solution of respective samples (GelMA (10 wt %), PEGDA (10 wt %), and LAP (0.3 wt %)) were added into a specific cylindric mold (8 mm in diameter and 1 mm thickness) and placed under UV light (360–480 nm, 6.9 mW/cm^2^) for 5 min. The cylindrical discs were further immersed in PBS solution for 24 h at 37 °C to reach swelling equilibrium. Subsequently, the swollen hydrogels samples were subjected to an unconfined compression test at a rate of 1 mm/min using a texture analyzer (TA.XT plus, Stable Micro Systems Ltd., Vienna, UK). The compressive modulus was determined as the slop linear region in the 0%–10% strain range of the stress–strain curve. All experiments were conducted in triplicate. To statistical test one-way ANOVA followed by post-test multiple Tukey comparisons were used. *p* < 0.05 was used to define the significance.

## 3. Results and Discussions

**Figure 2:**
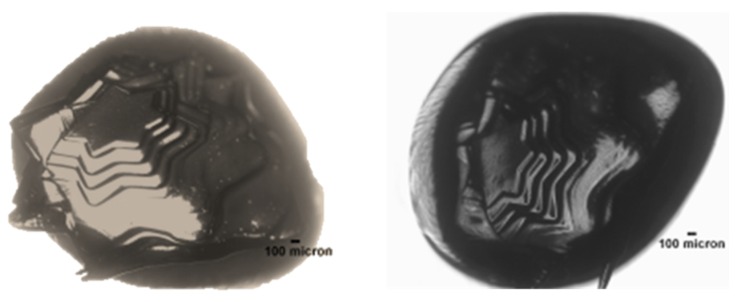
Star-like constructions obtained in 7 layers using PEGDA (10 wt %) and LAP (0.5 wt %).

The stereolithography technology was used to create PEGDA and GelMA hydrogel structures with channels of varying shapes and sizes. The desired configurations and structures of various shapes were designed by computer. Initially, as demonstrated in Figure 2, a star-like construct could be devised using PEGDA (10 wt %) and the photoinitiator (LAP, 0.3 wt %). The PEGDA hydrogel changed into a solid layer at the laser-irradiated site, then moved further downward (*z*-axis), and a new layer of hydrogel was obtained.

**Figure 3:**
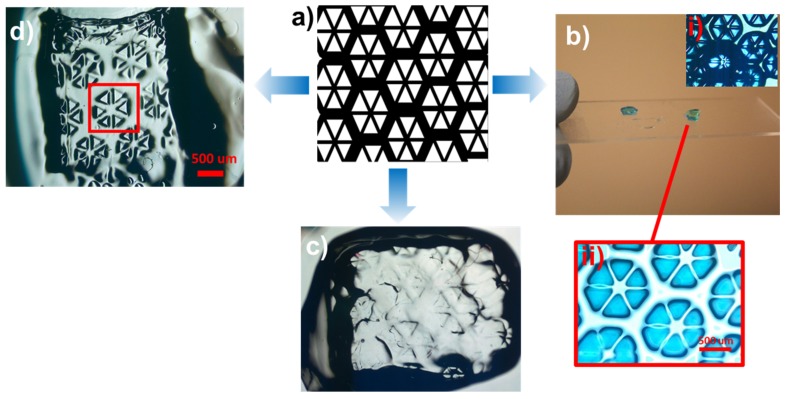
Printed model structures using PEGDA and GelMA hydrogels. (**a**) The computer-aided design (CAD) model to be printed. (**b**) Printed three-layered structure from PEGDA (10 wt %) and (i), (ii) the zoomed images. (**c**) The model printed with 10 wt % GelMA and (**d**) with 20 wt % GelMA.

Additionally, Figure 3a depicts the printing of complex structures from an input drawing. Using this mask, a 3D structure could be designed with PEGDA (10 wt %) and LAP (0.3 wt %) with three layers, providing figures with a diameter of 1 mm (Figure 3b). Subsequently, a hydrogel structure containing GelMA (10 wt %) was printed. These examples highlight the ability to construct and print 2- and 3D structures by using this technology and PEGDA and GelMA as the light-sensitive polymer materials. Interestingly, employing 20 wt % GelMA provided a printed construct with improved edge resolution compared to 10 wt % GelMA (Figure 3c,d).

**Figure 4:**
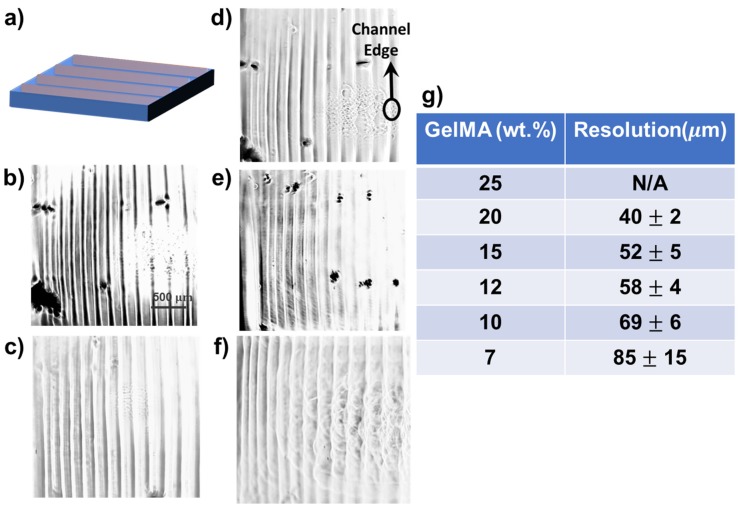
Printed channels using different concentrations of GelMA. (**a**) The input image consisting of two layers. The printed structure with (**b**) 20 wt % GelMA, (**c**) 15 wt % GelMA, **(d**) 12 wt % GelMA, (**e**) 10 wt % GelMA, and (**f**) 7 wt % GelMA. (**g**) The resolution analysis performed using ImageJ.

Previous studies have demonstrated that GelMA displays good biocompatibility, but insufficient mechanical properties, and its high viscosity limits its application in inkjet bioprinting [25,26]. Nevertheless, through combinations with other materials such as alginate, its mechanical properties can be tuned to provide well-organized encapsulated cells, human mesenchymal stem cells, and endothelial cells [27]. Figure 4a demonstrates the input image with two layers. We also demonstrated the successful printing of various channels with good distribution and extruded edges employing 20% (Figure 4b), 15% (Figure 4c), 12% (Figure 4d), 10% (Figure 4e), and 7% (Figure 4f) GelMA and their x–y-planes resolution analysis using ImageJ (Figure 4g). Note that 25 wt % GelMA did not deliver any desired printed structure; thus, the concentration was too high to provide a printable polymer solution. These analyses indicated that the edge resolution increased as the GelMA concentration increased (Figure 4g). A previous study demonstrated that GelMA concentration between 7 and 15 wt % could provide printed cell-laden constructs [28]. However, in the present study, layers with uniform and structured edges were designed.

**Figure 5:**
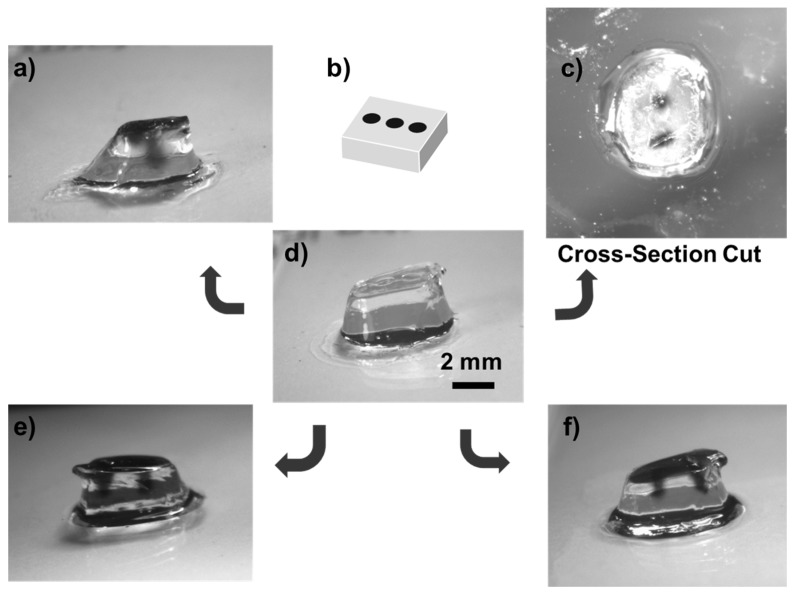
Printed 3D microchannels and microfluidic structures employing GelMA (10 wt %). (**a**) The 3D printed structure with 10 wt.% GelMA. (**b**) The mask employed to print the 3D structures. (**c**) 3D structure with vascularity. (**d**) printed structure and its transverse section. (**e**), (**f**) Printed structures with microstructures and microchannels.

Furthermore, Figure 5a illustrates the 3D printed structure using 10 wt % GelMA hydrogel and Figure 5b a mask used to print the 3D structures. Figure 5c shows the 3D structure with vascularity due to channels formed in the *z*-direction, resulting from the presence of dark liquid. Moreover, Figure 5d illustrates the structure after printing and its transverse section. The perfused printed structures are in Figure 5e,f. The printed models displayed microstructures and microchannels suitable for tissue engineering and drug delivery applications using the presented low-cost stereolithography.

**Figure 6:**
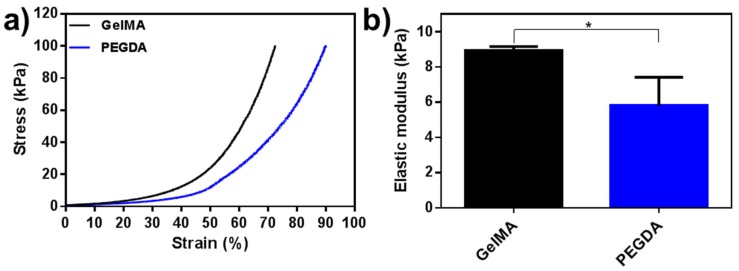
The mechanical evaluation of the printed GelMA and PEGDA hydrogels. (**a**) Strain–stress curves of the two hydrogels. (**b**) The compressive Young’s modulus of the GelMA and PEGDA hydrogels. The results were presented as mean and standard deviation. Statistical test: one-way ANOVA followed by post-test multiple Tukey comparisons. * *p* < 0.05 (N = 3).

The mechanical properties of the crosslinked GelMA and PEGDA hydrogels were further evaluated (Figure 6a,b). The PEGDA hydrogel displayed higher compressive stress and strain than the GelMA hydrogel, which indicated that a lower concentration could be used for printing (Figure 6a). The compressive Young’s modulus of GelMA and PEGDA were 8.72 and 5.70 kPa, respectively (Figure 6b). The GelMA hydrogel presented relatively “soft” behavior, and based on the mechanical results, the printability and resolution can be improved by the addition of PEGDA for the engineering of GelMA composite constructs.

The devised technology presents advancements compared to previous reports that employ microfluidic systems to manufacture drug carriers or direct delivery of drugs to a target tissue [29]. Other examples have divulged the use of 3D printed microfluidic chips as controllable 3D cell culture scaffolds, and presented the applicability of the technique in physiological systems for future bioengineering applications [30]. Our new technology is easily modulated to print different structures and different channels to construct microbioreactors.

## 4. Conclusions

The home-made low-cost stereolithography technology with a projector allowed the successful construction of 3D structures. The devised technique provides faster construction, higher resolution, and material conservation. Multi-layers, 3D, and vascularized structures were successfully obtained using PEGDA and GelMA as the polymers. We also investigated the concentration of GelMA as a strategy to obtain high-resolution channels with interesting resolutions. The new technology could be used to develop microbioreactors and opens up the possibility of manufacturing miniaturized microchannels and vascularized structures suitable as cell models for studies of pathologies, cell differentiation, or drug interactions. We envision that the presented technology will advance the research field of developing technologies for printing 3D hydrogel structures.
